# Vocal recognition of a nest-predator in black grouse

**DOI:** 10.7717/peerj.6533

**Published:** 2019-03-15

**Authors:** Richard Policht, Vlastimil Hart, Denis Goncharov, Peter Surový, Vladimír Hanzal, Jaroslav Červený, Hynek Burda

**Affiliations:** 1 Department of Game Management and Wildlife Biology, Faculty of Forestry and Wood Sciences, Czech University of Life Sciences Prague, Czech Republic; 2 Department of Forest Management, Faculty of Forestry and Wood Sciences, Czech University of Life Sciences Prague, Czech Republic

**Keywords:** *Tetrao*, Warning call, Corvids, Vocal recognition, Nest predator, Playback, Acoustic, Predation

## Abstract

Corvids count among the important predators of bird nests. They are vocal animals and one can expect that birds threatened by their predation, such as black grouse, are sensitive to and recognize their calls. Within the framework of field studies, we noticed that adult black grouse were alerted by raven calls during periods outside the breeding season. Since black grouse are large, extremely precocial birds, this reaction can hardly be explained by sensitization specifically to the threat of nest predation by ravens. This surprising observation prompted us to study the phenomenon more systematically. According to our knowledge, the response of birds to corvid vocalization has been studied in altricial birds only. We tested whether the black grouse distinguishes and responds specifically to playback calls of the common raven. Black grouse recognized raven calls and were alerted, displaying typical neck stretching, followed by head scanning, and eventual escape. Surprisingly, males tended to react faster and exhibited a longer duration of vigilance behavior compared to females. Although raven calls are recognized by adult black grouse out of the nesting period, they are not directly endangered by the raven. We speculate that the responsiveness of adult grouse to raven calls might be explained as a learned response in juveniles from nesting hens that is then preserved in adults, or by a known association between the raven and the red fox. In that case, calls of the raven would be rather interpreted as a warning signal of probable proximity of the red fox.

## Introduction

Fragmentation of extensive forests in Europe is assumed to be the key factor causing population decline of tetraonid grouse (Åhlen et al., 2013; Kurki et al., 2000; Storch, 2007). Changes in habitat structure and composition of predators are known to interact and affect predation pressure on birds (Fletcher et al., 2010; Van der Wal & Palmer, 2008). Widespread habitat fragmentation was accompanied by an increase in local populations of mesopredators including foxes and nest predating corvids (Andren, 1992; Kurki et al., 2000). Ground-nesting birds are particularly susceptible to predation both by mammalian and avian predators, and not only on incubating females, but also eggs and chicks (Fletcher et al., 2010; Newton, 1993; Sullivan & Dinsmore, 1990). Goshawk and red fox were identified as two main predators of adult black grouse (Angelstam, 1984). Fox predation plays a significant role in grouse mortality, especially during winter (Kidawa & Kowalczyk, 2011). Nest predation was recognized as the most important direct cause of nest loss in grouse (Ludwig et al., 2010). Indeed, after an experimental corvid removal study, black grouse nest loss decreased (Parker, 1984). Nevertheless, compensatory nest predation by other predators has occurred in the absence of corvids. The complexity of the predator–prey relationships is evident in a study showing that the goshawk, representing the main predator of adult black grouse, might in turn provide a protective advantage for grouse, as goshawks are known to prey on corvids (Tornberg et al., 2016).

By chance, within the framework of ongoing bioacoustic field experiments, we noticed that adult male black grouse (*Tetrao tetrix*), appeared to react to playback calls of the raven during periods outside the breeding season. This was unexpected and prompted us to perform further experiments under controlled conditions to study the phenomenon systematically.

Black grouse nest on the ground with only hens incubating and rearing chicks. Chicks of tetraonids are extremely precocial; they follow the mother immediately after they have dried out and they develop rapidly, and are thus able to escape predators by flight just days after birth (De Juana, 1994). Given that eggs and chicks of ground nesting birds are threatened by nest predators, their parents are expected to be able to recognize and cope with the predation risk. Indeed, mammals and birds are known to react to predator calls, even if the respective predators do not vocalize during hunting (Blumstein et al., 2008).

We tested the hypothesis that black grouse recognize common ravens as a threat via their calls alone. We predicted that black grouse would exhibit greater alarm, as indicated by behavioral reactions, during playbacks of raven calls compared to playbacks of calls of nonthreatening, sympatric species.

If black grouse have a lower probability of misidentifying potentially threatening species when hearing calls of nonthreatening birds, grouse responsiveness should be similar towards calls of different control birds but different towards potentially threatening calls.

## Materials & Methods

### Ethics statement

The research was conducted in accordance with the guidelines of the Animal Behaviour Society for the ethical use of animals in research. The study was carried out in accordance with the recommendations in the Guide for Care and Use of Animals of the Czech University of Life Sciences Prague. The protocol was approved by the Animal Care and Use Committee of the Czech Ministry of the Environment (Permit number: 15106/ENV/14-825/630/14. We point out that every animal was provoked only once, that the disturbance did not exceed naturally occurring stimulation, and that the studied species is considered a game species in Finland. This project was carried out within the framework of the bilateral cooperation of the Czech Republic (Czech University of Life Sciences Prague) and Finland concerning game management under auspices of the Department of Natural Resources, Ministry of Agriculture and Forestry, Finland. According to Finnish legislation in general and to the hunting legislation, this type of scientific project does not require any special permits or licenses.

### Field tests

We recorded and analyzed reactions to playback calls of the common raven (a potential nest predator) and control (harmless) bird species. The study was conducted at three localities in Finland: Hollola (61°05′51.5″N 25°22′59.0″E), Kainuu (63°51′06.2″N 29°09′55.2″E), and Jäkäläkangas (63°59′30.4″N 26°53′26.6″E) during two lek seasons (2012 and 2013) in April–May. Each focal individual was tested only once (for both raven and control sound). When more individuals were present, we focused on the one individual that was most visible throughout the entire experimental session. For each session, behavior, sex, habitat (Open, Tree, Road edge) and distance from the speaker, time, weather conditions, and GPS coordinates were recorded.

**Figure 1:**
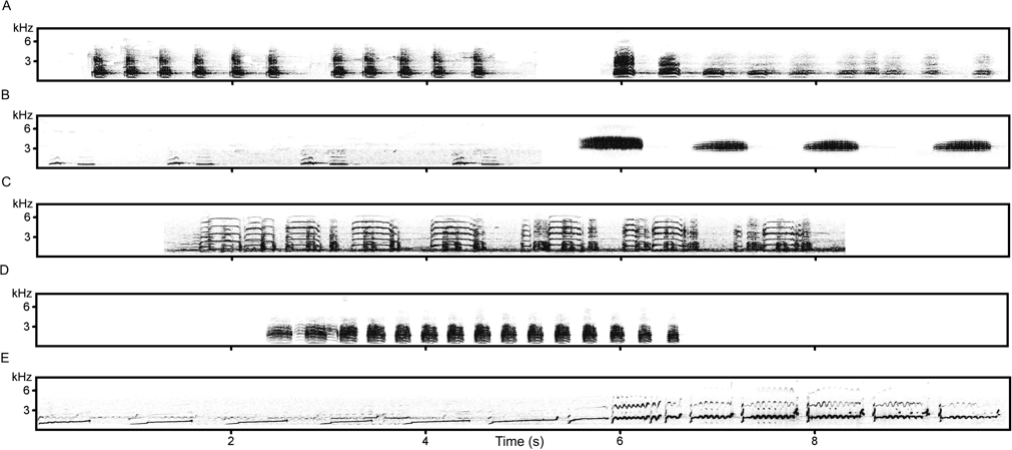
Spectrograms of calls used in playbacks. (A) Two playback versions of the Common raven: two series of calls (six and five calls) and one series containing ten calls. (B) Common cuckoo and Brambling. (C) Common crane. (D) Mallard. (E) The Eurasian curlew.

Each playback session consisted of calls of control bird species and the long-distance call frequently produced during flight by the common raven (*Corvus corax*) (Bergman & Helb, 1982). For the control sound, we used calls of five sympatric bird species commonly heard in the study area: brambling (*Fringilla montifringilla*), common crane (*Grus grus*), common cuckoo (*Cuculus canorus*), the Eurasian curlew (*Numenius arquata*) and mallard (*Anas platyrhynchos*), (Fig. 1). These were selected because black grouse regularly heard these calls during the observation period and their presence does not indicate increased risk of predation, nor any threat from a possible foraging competitor. They represent long-distance signals produced in non-alarming contexts. For bramblings, we used loud, sharp nasal calls that are produced frequently by males during the breeding season (Clement & Arkhipov, 2018). For common cranes, we used duet calls which are produced in sexual, territorial, and contact context when males utter a loud trumpeting call immediately followed by a lower-pitched female call (Archibald et al., 2018; Policht & Ticháčková, 2010). For common cuckoo, we used a male song, the characteristic vocalization of this species (Li et al., 2017; Payne, Christie & Kirwan, 2018). For the Eurasian curlew, we used calls that we recorded during context of meeting of both partners. For the mallard, we used one of the most familiar vocalizations, decrescendo of quacks which are frequently elicited from unmated females and from separated females (Abraham, 1974; Lorenz, 2011). We prepared five versions of the common raven playback and three playback versions of each control species. For each playback version (both for common raven and controls) calls from different individuals were selected. Stimuli used in raven playbacks were 4.1–4.5 s in duration and contained 10–12 calls, similarly brambling (4.2–4.4 s, 4 calls), common crane (6.3–6.5 s, one repetitive call), common cuckoo (4.6–4.8 s, 4 calls), the Eurasian curlew (9.8–10.0 s, one repetitive call) and mallard (4.2–4.6 s, 15–17 calls) (Fig. 1A). Instead of aligning the exact playback length, we decided not to edit inter-call intervals but rather kept their natural call length when creating the stimuli. Calls of these control bird species were obtained both from a commercial CD (all the bird songs of Britain and Europe, Roché, 1993, France) and calls recorded locally in the study area. Sound levels of all playback calls were standardized using root mean square in Avisoft software (Avisoft Bioacoustics, R. Specht, Berlin, Germany).

The session included one min pre-playback monitoring and one min post-playback observation which started by broadcasting the playback. The order of playbacks was randomized and the second playback followed approximately 2 min after the first playback and/or when the focal animal switched to a relaxed behavior. Such a matched pair design holds the environmental variables constant (Kroodsma, 1989). Tested sounds were played through a MIPRO MA-202 sound loudspeaker linked to Olympus PCM-11. Playbacks were played at peak sound pressure levels of about 93 dB at 1 m (measured by sound level-meter Voltcraft SL-200). Playback experiments were videotaped using a Canon digital camera (LEGRIA FS306) and the researcher remained hidden behind trees from a distance from 45 ± 16 m (mean ± SD). When we met the birds near the road, we stopped and conducted an experiment directly from the car not to prevent any disturbance. We started the playback after a 5–15 min habituation period waiting until they returned to their previous activity.

We travelled through the area in order to find groups of grouse on leks or solitary birds. To minimize the possibility of repeated testing of the same individuals, trials were separated by at least 3 km within and between test days. Although the black grouse were not marked, high population numbers and our experimental design minimized the probability of recording the same individuals (Carrasco & Blumstein, 2012; Martínez & Zenil, 2012). We consider such design to be sufficient because this grouse is considered to be largely sedentary with limited daily movements (De Juana, 1994) and males show limited interactions with males from other leks. They are recruited on the lek locally with a high probability to remain on the same lek until the following year (Borecha, Willebrand & Nielsen, 2017).

### Data analysis

We measured duration and latency of the response using frame by frame analysis of the video records 15 s before and after the onset of corvid and control sounds. Latency of the response was measured from the beginning of playback. We categorized behavioral reactions as follows: (1) Scanning (scanning their surroundings), (2) Strong vigilance (initiated with neck stretching, followed by scanning or escaping). Only completed experiments were used in the analyses. We did not include playbacks when the focal bird disappeared from sight or when any disturbance occurred during the experiment. We used Principal Component Analysis (PCA) to express the overall response, where the individual PC factors were used as response scores of the black grouse’ response to playbacks.

We used non-parametric tests: Wilcoxon Matched Pairs Tests for paired comparisons of responses to particular species-specific sounds, Mann–Whitney U Tests and Kruskal-Wallis Tests for comparing independent samples. These tests were performed with software Statistica 12.0 (StatSoft Inc., Tulsa, OK, USA). The three variables: duration, latency and intensity each describes a component of the reaction to the playback stimuli. Descriptive statistics include mean ± SE. The Generalized Linear Mixed Model (GLMM) in IBM SPSS 23 was used for evaluation of a potential effect of habitat, sex, number of birds and distance on overall response (PC1).

## Results

**Figure 2:**
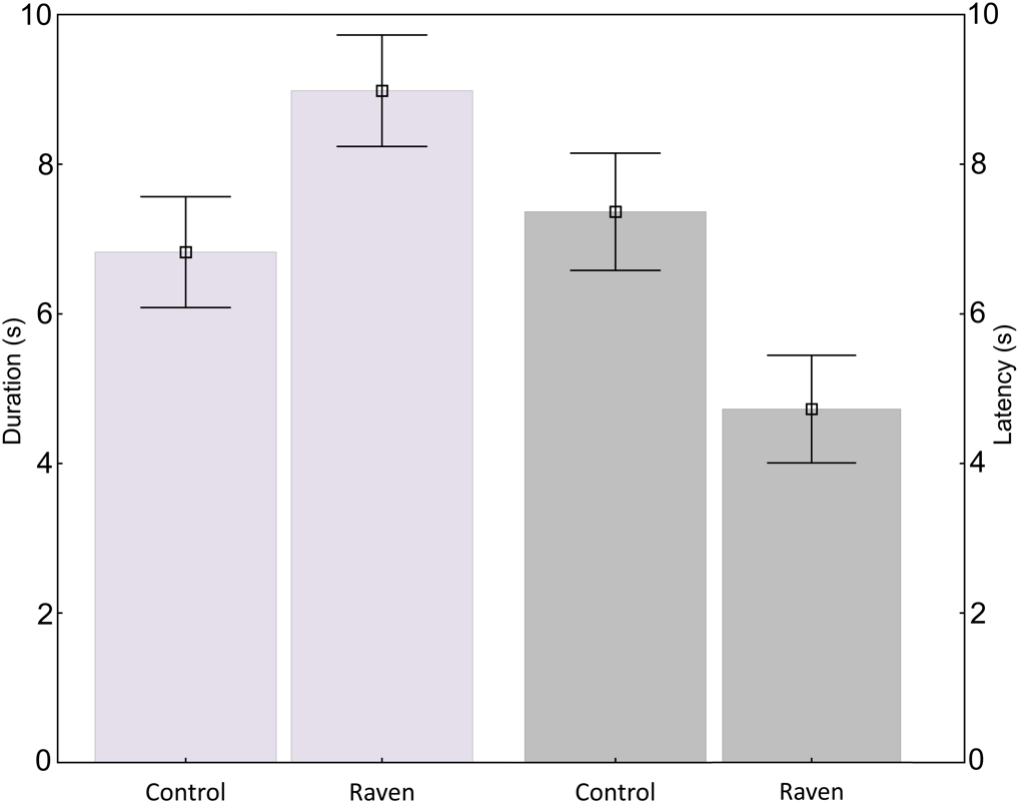
Duration and latency of the response. Black grouse responded to calls of the common raven in shorter latency and their reactions lasted a longer time in comparison to calls of non-corvid birds. Bars show mean with SE.

We found that black grouse frequently exhibited vigilance shortly after hearing playback of the common raven. During the 63 trials with playback of raven calls, the majority of subjects (71%) showed an intense response, with stretched neck, scanning, or escape, Wilcoxon Matched Pairs Test: *p* < 0.001. Such a response was recorded neither during the pre-playback period nor after control calls. Black grouse were able to recognize calls of the common raven from those of control bird species. Focal birds responded to calls of the common raven faster (with shorter latency) in comparison to calls of non-corvid birds (Wilcoxon Matched Pairs Test: *p* = 0.001). Latency of the response to control stimuli lasted 7.37 ± 0.78 s (mean ± SE), while to call of common raven 4.81 ± 0.73 s. Duration of the response to nonthreating control stimuli was 6.83 ± 0.74 s and to the raven call was 8.98 ± 0.74 s (Fig. 2) and differed significantly (Wilcoxon Matched Pairs Test: *p* = 0.001).

Black grouse responded with increased vigilance and/or escape more frequently to raven calls in comparison to vocalization of non-predatory birds, suggesting increased vigilance to perceived predation risk. Responses of males were more intensive than those of females (*p* = 0.039; Chi-Square), were longer (*p* = 0.002, Mann–Whitney U Test) and had a shorter latency (*p* = 0.024, Mann–Whitney U Test).

To express the overall response, the individual PC factors (response scores) were used as a component measure of the black grouse’ response to playbacks. The PCA revealed two principal components with an eigenvalue >1 explaining 63% of the variation. Because both duration and latency of the response (both were Z transformed) mostly correlated with PC1 (duration: *r* =  − 0.94, latency: *r* = 0.93), individual PC1 factors were used for the following testing. Principal component analysis revealed highly significant response differences to the playback of raven calls compared to the calls from control species (Wilcoxon Matched Pairs Test: *p* < 0.001). Responses to different versions of control stimuli did not differ: Kruskal-Wallis Test: H (5, *n* = 63) =9,3; *p* = 0.097.

Evaluation of following variables on overall response (PC1) included habitat, sex, number of birds and distance. Normal distribution of errors with Identity link function of GLMM (*F* = 6.54; *df*1 = 5, *df*2 = 56, *p* < 0.001) revealed only sex to have significant influence on overall responses (*F* = 13.6; *df*1 = 1, *df*2 = 56, *p* = 0.001), while the other variables including interactions were not significant.

## Discussion

Our study demonstrates that black grouse are able to distinguish calls of the common raven, a potential nest predator, from calls of non-predatory birds. Since the common raven is a vocal bird, timely reactions of black grouse to these signals, coupled with adaptive behavioral responses, such as freezing or hiding, could minimize the predation risk of the nest with eggs or chicks.

The manner and intensity with which a potential prey responds to predators depends on the ability to assess the level of predation risk and to distinguish between nonthreatening and threatening signals (Adams et al., 2006). We showed that the raven is recognized by adult grouse which are actually not directly threatened by corvids. Moreover, we recorded alert responses during periods outside the breeding season, and surprisingly males, who do not provide any parental care to chicks on the nest, reacted more strongly than females. Although the females also responded outside the breeding season, the mating period represents the period immediately preceding the nesting period. Therefore, females could be theoretically motivated to the behavior that will follow soon after mating. More robust male responses compared to that of females could reflect sexually dimorphic anti-predatory strategies. Less robust female responses could mirror the more frequent motionless camouflage response, a typical female behavioral phenotype, especially during nesting.

Potential sensitivity and specific reactivity to raven calls may be innate (analogous to responses of chicks belonging to diverse fowl species to silhouettes of raptors, cf. (Tinbergen, 1951), and the responses may be preserved throughout life. The alternative (or complementary) explanation of the responses of adult black grouse to calls of ravens could be seen in the possibility that the black grouse perceive the raven call as a non-specific signal informing about danger, e.g., presence of other predators such as the red fox or humans. Particularly, the association between the common raven and the red fox is well known and a fox could follow calls of ravens to pilot to carcasses while ravens provide alarm calls after sighting a fox (Killengreen et al., 2012; Selva et al., 2003). Thus, calling ravens linked with increased probability of the presence of predators may evoke adaptive anti-predator responses in adult grouse of both sexes. Similarly, ravens are scavengers, closely associated with large carnivores (Marzluff, 2018), and have been shown to be attracted by wolf howling (Harrington, 1978). Although, the impact of corvids on breeding failure has been intensively studied in many bird species, the response of birds to corvid vocalization has been a subject of relatively few studies, and focused only on altricial birds (cf. Eggers, Griesser & Ekman, 2005; Eggers et al., 2006).

Predation represents a major cause of breeding failure in many bird species (Ricklefs, 1969). Corvids are generalist predators and exploit areas inhabited by grouse and altered by humans (Manzer & Hannon, 2005), particularly in fragmented landscapes (Haegen, Schroeder & DeGraaf, 2002). In the black grouse, predation has been recognized as a principal proximate cause of mortality and breeding failure, where the red fox, mustelids, and corvids represent the main predators of eggs and chicks (Angelstam, 1984; Caizergues & Ellison, 1997; Parker, 1984; Willebrand & Marcström, 1988), whereas raptors, especially the goshawk, predate adult grouse (Caizergues & Ellison, 2000; Tornberg, 2001). To our knowledge, this study is the first to show that a precocial bird species responds to calls of a nest predator. Such ability could represent an additional behavioral adaptation against nest predation. Another known strategy used by grouse species includes camouflage hen color including inactive behavior during incubation, escape by flight, grouping in flocks (Angelstam, 1984), selection of nest and brood-rearing sites with lower predator densities (Dinkins et al., 2012), and similarly, switching nest sites after nest predation (Marjakangas, Valkeajärvi & Ijäs, 1997). One possibility is that hens evaluate the potential safety of nest sites based on the acoustic landscape on the local area. Expression of this ability and recognition of calling ravens could be learned by chicks resulting in anti-predator reactions in mature adults of both sexes. To resolve these possibilities, further studies are needed on precocial birds.

## Conclusions

Although predation by avian predators, such as corvids, on nests ranks among the major causes of breeding failure in birds, recognition of predator vocalizations remains poorly studied, especially in precocial species. We tested whether the black grouse (*Tetrao tetrix*) are able to distinguish calls of the common raven, a potential nest predator, from those of sympatric and harmless bird species, representing a control group: Eurasian curlew, brambling, common crane, mallard and common cuckoo.

We show that black grouse can distinguish between calls of the common raven, a potential nest predator, and the nonthreatening birds tested. Responses of males were more pronounced compared to females. Since corvid nest predation is more likely to occur on eggs rather than precocial chicks, the study was done prior to the nesting period. Because males are not involved in brood care and adults are not directly threatened by corvids, the question raised is what is the adaptive significant of specific alert responses exhibited my male black grouse to raven calls?

Such an ability might be learned by chicks from the female, and if so, a learned reaction would then continue to mature in both sexes. We also speculate that calling signals from the common raven could inform grouse about the presence and proximity of larger predators, particularly red foxes. Such hypotheses need to be directly tested. According to our knowledge, this is the first evidence for the ability of a precocial bird species to respond to vocalizations from a nest-predator.

##  Supplemental Information

10.7717/peerj.6533/supp-1 Supplemental Information 1 Measured data from playback experiments (Sex) Sex of tested black grouse. (Habitat) Habitat of tested black grouse. (Nu_birds) Number of birds. (Distance) Distance to tested individual. (Contr_Dur) Response duration to control. (Contr_Lat) Response latency to control. (Contr_Int) Response intensity to control. (Contr_PC1) First principal component generated by PCA in control. (Raven_Dur) Response duration to Raven. (Raven_Lat) Response latency to Raven. (Raven_Int) Response intensity to Raven. (Raven_PC1) First principal component generated by PCA in Raven.

10.7717/peerj.6533/supp-2 Supplemental Information 2 Playback experiment example Response to playback of the common crane as a control sound and common raven.

## Additional Information and Declarations

### Data Availability

The following information was supplied regarding data availability: The raw data are available in the Supplemental Files.
