# Supplementary material for: Vocal recognition of a nest-predator in black grouse
Source: PeerJ. 2019 Mar 15;7:e6533. doi: 10.7717/peerj.6533 (PMC6422015; doi:10.7717/peerj.6533)
Supplement: Supplemental Information 1 — (Sex) Sex of tested black grouse. (Habitat) Habitat of tested black grouse. (Nu_birds) Number of birds. (Distance) Distance to tested individual. (Contr_Dur) Response duration to control. (Contr_Lat) Response latency to control. (Contr_Int) Response intensity to control. (Contr_PC1) First principal component generated by PCA in control. (Raven_Dur) Response duration to Raven. (Raven_Lat) Response latency to Raven. (Raven_Int) Response intensity to Raven. (Raven_PC1) First principal component generated by PCA in Raven. [file peerj-07-6533-s001.docx]

| Sex | Habitat | Nu_birds | Distance | Contr_Dur | Contr_Lat | Control_Int | Contr_PC1 | Raven_Dur | Raven_Lat | Raven_Int | Raven_PC1 |
| --- | --- | --- | --- | --- | --- | --- | --- | --- | --- | --- | --- |
| male | open | 2 | 30 | 0 | 15 | 1 | 1.144910 | 13 | 2 | 2 | -1.049765 |
| male | open | 3 | 30 | 10 | 2 | 1 | -0.534470 | 14 | 1 | 2 | -1.203389 |
| male | tree | 1 | 30 | 14 | 1 | 1 | -0.895630 | 14 | 1 | 2 | -1.195514 |
| male | tree | 7 | 50 | 10 | 4 | 1 | -0.391820 | 9 | 1 | 2 | -0.831250 |
| female | road edge | 1 | 30 | 13 | 2 | 1 | -0.257008 | 14 | 1 | 2 | -0.702640 |
| female | tree | 1 | 40 | 14 | 1 | 1 | -0.402757 | 14 | 1 | 1 | -0.702640 |
| female | open | 1 | 30 | 0 | 15 | 1 | 1.637784 | 0 | 6 | 2 | 0.695973 |
| male | open | 1 | 70 | 12 | 3 | 1 | -0.604124 | 6 | 3 | 2 | -0.457442 |
| female | open | 1 | 50 | 0 | 15 | 1 | 1.637784 | 13 | 2 | 2 | -0.556892 |
| female | open | 1 | 50 | 0 | 15 | 1 | 1.629908 | 0 | 15 | 1 | 1.330025 |
| male | road edge | 5 | 30 | 14 | 1 | 1 | -0.895630 | 13 | 2 | 2 | -1.049765 |
| female | tree | 1 | 30 | 11 | 4 | 1 | 0.026623 | 14 | 1 | 1 | -0.710516 |
| male | tree | 1 | 30 | 14 | 1 | 1 | -0.895630 | 14 | 1 | 2 | -1.195514 |
| male | tree | 1 | 30 | 10 | 5 | 1 | -0.320493 | 13 | 2 | 2 | -1.057640 |
| male | tree | 1 | 50 | 13 | 2 | 1 | -0.757757 | 13 | 2 | 2 | -1.057640 |
| male | tree | 1 | 50 | 8 | 6 | 1 | -0.100318 | 14 | 1 | 1 | -1.203389 |
| female | road edge | 3 | 30 | 14 | 1 | 1 | -0.410632 | 14 | 1 | 2 | -0.710516 |
| female | road edge | 1 | 30 | 0 | 15 | 1 | 1.641721 | 0 | 15 | 1 | 1.341838 |
| female | road edge | 1 | 30 | 0 | 15 | 1 | 1.629908 | 0 | 15 | 1 | 1.330025 |
| female | road edge | 1 | 30 | 0 | 15 | 1 | 1.629908 | 6 | 1 | 2 | -0.115095 |
| female | road edge | 1 | 30 | 0 | 15 | 1 | 1.629908 | 5 | 1 | 2 | -0.040664 |
| female | road edge | 1 | 30 | 0 | 15 | 1 | 1.629908 | 0 | 15 | 1 | 1.330025 |
| female | road edge | 1 | 30 | 0 | 15 | 1 | 1.629908 | 0 | 15 | 1 | 1.330025 |
| male | open | 4 | 50 | 7 | 8 | 1 | 0.124638 | 13 | 2 | 2 | -1.049765 |
| male | open | 1 | 50 | 10 | 5 | 1 | -0.308680 | 0 | 15 | 1 | 0.848965 |
| female | road edge | 1 | 30 | 0 | 15 | 1 | 1.637784 | 13 | 2 | 2 | -0.556892 |
| female | road edge | 1 | 30 | 3 | 1 | 1 | 0.415946 | 8 | 1 | 2 | -0.256076 |
| female | road edge | 1 | 30 | 0 | 15 | 1 | 1.629908 | 0 | 15 | 1 | 1.330025 |
| female | road edge | 1 | 30 | 8 | 6 | 1 | 0.400431 | 0 | 15 | 1 | 1.337900 |
| female | open | 1 | 70 | 5 | 4 | 1 | 0.481067 | 0 | 15 | 1 | 1.337900 |
| female | road edge | 1 | 20 | 0 | 15 | 1 | 1.629908 | 0 | 15 | 1 | 1.330025 |
| female | road edge | 1 | 30 | 0 | 15 | 1 | 1.629908 | 0 | 15 | 1 | 1.330025 |
| female | road edge | 1 | 30 | 14 | 1 | 1 | -0.398820 | 3 | 1 | 2 | 0.120000 |
| female | road edge | 1 | 30 | 0 | 15 | 1 | 1.629908 | 12 | 3 | 2 | -0.419009 |
| female | road edge | 1 | 30 | 2 | 1 | 1 | 0.490377 | 14 | 1 | 2 | -0.702640 |
| female | road edge | 1 | 30 | 0 | 15 | 1 | 1.629908 | 11 | 4 | 2 | -0.273260 |
| female | road edge | 1 | 30 | 13 | 2 | 1 | -0.264883 | 12 | 3 | 2 | -0.419009 |
| female | road edge | 1 | 50 | 13 | 2 | 1 | -0.253071 | 13 | 2 | 2 | -0.552954 |
| male | open | 3 | 50 | 0 | 15 | 1 | 1.137035 | 13 | 2 | 2 | -1.057640 |
| female | road edge | 1 | 30 | 0 | 15 | 1 | 1.641721 | 0 | 15 | 1 | 1.341838 |
| female | road edge | 2 | 30 | 14 | 1 | 1 | -0.402757 | 14 | 1 | 2 | -0.702640 |
| male | open | 2 | 50 | 0 | 15 | 1 | 1.144910 | 13 | 2 | 2 | -1.049765 |
| male | open | 2 | 50 | 13 | 2 | 1 | -0.757757 | 13 | 2 | 2 | -1.057640 |
| male | open | 1 | 50 | 15 | 15 | 1 | 0.028495 | 10 | 1 | 2 | -0.897801 |
| male | road edge | 1 | 50 | 11 | 2 | 1 | -0.604963 | 14 | 1 | 2 | -1.199451 |
| male | open | 2 | 50 | 0 | 15 | 1 | 1.133098 | 0 | 15 | 1 | 0.833214 |
| male | open | 1 | 50 | 15 | 1 | 1 | -0.981869 | 14 | 1 | 2 | -1.207326 |
| male | road edge | 1 | 50 | 0 | 15 | 1 | 1.140973 | 13 | 2 | 1 | -1.053702 |
| male | open | 2 | 70 | 10 | 2 | 1 | -0.530532 | 9 | 2 | 2 | -0.755990 |
| male | open | 1 | 70 | 13 | 2 | 1 | -0.753819 | 14 | 1 | 2 | -1.199451 |
| male | open | 2 | 80 | 0 | 15 | 1 | 1.133098 | 14 | 1 | 1 | -1.207326 |
| male | open | 6 | 70 | 6 | 2 | 1 | -0.228886 | 7 | 1 | 2 | -0.674519 |
| male | tree | 1 | 70 | 10 | 4 | 1 | -0.380007 | 14 | 1 | 2 | -1.191576 |
| male | tree | 1 | 80 | 8 | 7 | 1 | -0.032928 | 14 | 1 | 2 | -1.207326 |
| male | road edge | 1 | 60 | 6 | 2 | 1 | -0.228886 | 11 | 1 | 2 | -0.972231 |
| female | tree | 1 | 70 | 14 | 1 | 1 | -0.414570 | 14 | 1 | 2 | -0.714453 |
| male | open | 1 | 70 | 10 | 2 | 1 | -0.526595 | 0 | 15 | 1 | 0.845027 |
| male | open | 2 | 70 | 9 | 1 | 1 | -0.535304 |  | 2 | 2 |  |
| male | road edge | 1 | 50 | 5 | 6 | 1 | 0.134782 | 14 | 1 | 2 | -1.191576 |
| male | tree | 1 | 50 | 0 | 15 | 1 | 1.148848 | 0 | 15 | 1 | 0.848965 |
| male | open | 2 | 70 | 13 | 2 | 1 | -0.761694 | 14 | 1 | 2 | -1.207326 |
| male | open | 1 | 50 | 13 | 2 | 1 | -0.749881 | 12 | 3 | 2 | -0.904007 |
| male | open | 1 | 70 | 13 | 2 | 1 | -0.761694 | 14 | 1 | 2 | -1.207326 |
